# Exploring the association of IL-10 polymorphisms in Behcet’s disease: a systematic review and meta-analysis

**DOI:** 10.1186/s12950-019-0230-2

**Published:** 2019-12-23

**Authors:** Elham Shahriyari, Leila Vahedi, Nasrin Roshanipour, Mohammad Asghari Jafarabadi, Amin Khamaneh, Maryam Ghaffari Laleh

**Affiliations:** 1 Center of Excellence for Biodiversity, Faculty of Natural Sciences, University of Tabriz, Tabriz, Iran; 2 Liver and Gastrointestinal Diseases Research Center, Tabriz University of Medical Sciences, Tabriz, Iran; 3 Department of Biology, School of Genetic, Azad University of Tabriz, Tabriz, Iran; 4 Department of Statistics and Epidemiology, Faculty of Health, Tabriz University of Medical Sciences, Tabriz, Iran; 5 Student Research Committee, Tabriz University of Medical Sciences, Tabriz, Iran

**Keywords:** Behçet’s disease, Interleukin-10, Polymorphism, Meta-analysis

## Abstract

**Background:**

Polymorphisms in the interleukin-10 (IL-10) gene have been studied in various ethnic groups for possible association with Behçet’s disease (BD). This study aimed to perform a meta-analysis of eligible studies to calculate the association of IL-10 polymorphisms with BD.

A systematic literature search was carried out in PubMed, Embase, Web of Science, and Scopus databases to identify relevant publications, and extracted the respective results. Pooled odds ratio (OR) with 95% confidence interval (CI) was used to evaluate the power of association with a random-effects model.

**Results:**

A total of 19 articles, consisting of 10,626 patients and 13,592 controls were included in the meta-analysis. The meta-analysis revealed significant associations in allelic and genotypic test models of − 819 (C vs. T: OR = 0.691, *P* < 0.001; CC vs. TT: OR = 0.466, *P* < 0.001; CC + CT vs. TT: OR = 0.692, *P* < 0.001; and CC vs. CT + TT: OR = 0.557, *P* < 0.001), − 592 (C vs. A: OR = 0.779, *P* = 0.002; CC + AA vs. AA: OR = 0.713, *P* = 0.021; and CA vs. AA: OR = 0.716, *P* = 0.016), rs1518111 (G vs. A: OR = 0.738, *P* < 0.001; GG vs. AA: OR = 0.570, *P* < 0.001; GG + AG vs. AA: OR = 0.697, *P* < 0.001; GG vs. GA + AA: OR = 0.701, *P* < 0.001; and AG vs. GG: OR = 0.786, *P* = 0.004) and rs1554286 (C vs. T: OR = 0.582, *P* < 0.001; CC vs. TT: OR = 0.508, *P* < 0.001; CC + CT vs. TT: OR = 0.605, *P* < 0.001; CC vs. CT + TT: OR = 0.665, *P* = 0.012; and CT vs. TT: OR = 0.646, *P* = 0.001). However, we failed to find any association between − 1082 polymorphism and susceptibility of BD.

**Conclusion:**

This meta-analysis demonstrated that the interleukin-10 -819, − 596, rs1518111 and rs1554286 polymorphisms could be responsible against BD susceptibility, and should probably be regarded as a protective factor for Behçet’s disease.

## Background

Behçet’s disease (BD) is a chronic remitting systemic vasculitis of unrevealed etiology [1]. BD is heterogeneous in onset [2] that its common clinical manifestations are orogenital ulcers, skin lesions, and uveitis [3]. BD is frequently observed in the Far East and the Middle East; however, it has distributed along the Old Silk Route due to immigration [1, 4–6]. Although the exact cause of Behçet’s disease is mostly indefinite, it is believed that the pivotal pathophysiologic event is an inflammatory reaction to environmental factors in a genetically predisposed host and presence of epigenetic changes [7–11]. Research has evidenced that various cytokine networks play a crucial role in the determination of the disease onset, course of the disease, and outcome [12]. Interleukin-10 (IL-10) [Gene ID: 3586] is a multifunctional cytokine that acts upon other cytokines’ signaling pathways [13]. IL-10 is a Type II cytokine in a family, including IL-19, IL-20, IL-22, IL-26, and IL-29 [14]. IL-10 is expressed mostly by activated monocytes/macrophages, natural killer cells, dendritic cells, mast cells, T lymphocytes (mainly Th2 subsets), and B lymphocytes [15].

This immuno-regulatory cytokine was demonstrated to have an imperative cytokine-suppressing role in the autoimmunity and inflammatory responses as a result of its ability to the downregulate antigen presentation and macrophage activation [16–20]. The abundant evidence has revealed that low IL-10 expression is involved in the pathogenesis of inflammatory and autoimmune diseases such as BD [21]. Furthermore, polymorphisms in multiple immuno-regulatory genes have indicated as a risk predisposition for the developing of BD owing to their effect on the cytokine production [22]. The human IL-10 gene maps to 1q31–32 and contains five exons and four introns. The IL-10 promoter is highly polymorphic and these single nucleotide polymorphisms (SNPs) appear to be correlated to the expression level of IL-10. Three frequently investigated SNPs of IL-10 gene are − 1082 A to G (rs1800896) -lies within a putative Ets transcription factor binding site-, − 819 T to C (rs1800871) -placed within a putative positive regulatory region-, and − 592 A to C (rs1800872) -is located within a putative STAT-3 binding site and negative regulatory region- polymorphisms [23, 24]. Rs1518111 (2195 A to G) which is in linkage disequilibrium (LD) with rs1800896 and rs1554286 (2607 T to C) that is in a strong LD with rs1800571 are the intronic variant of second and third exons [25, 26].

Considering the role of IL-10 in BD and the correlation between the IL-10 gene polymorphisms and IL-10 production, several studies have been carried out to investigate the association of the IL-10 gene polymorphisms with the BD susceptibility. However, contradictory reported results and/or small sample size could lead to low statistical power and false-positive results. Therefore, in order to deal with these ambiguities, and potential biases, such as publication bias and providing increased statistical power, we turned to meta-analysis. In the present study, a meta-analysis of all available published studies was carried out to clarify the association between the IL-10 gene polymorphisms and the BD susceptibility.

## Results

### Literature search and study characteristics

**Fig. 1:**
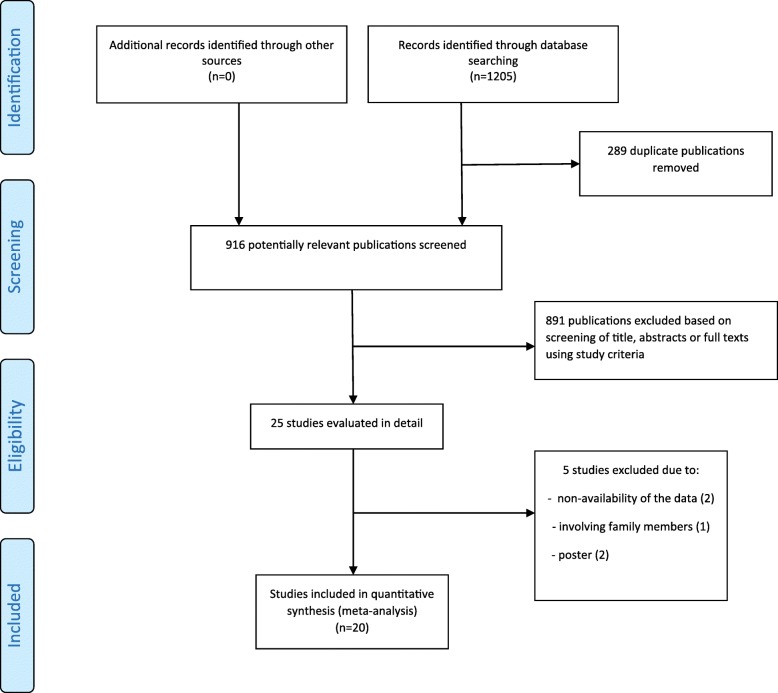
Flow diagram of studies for inclusion in the systematic review and meta-analysis

The literature search filtered 2390 English and Persian-language potential articles related to the IL-10 polymorphisms and BD. After the removal of duplicates, other publications were reviewed based on the titles and abstracts. In the end, 24 eligible studies were selected with a full-text review where 5 studies were eliminated (two studies due to non-availability of the data, even no responses could be gotten after having sought the relevant data via email contacts, two were posters and one study was involving family members). A flowchart of the study selection process and its results is presented in Fig. 1.

We conduct the meta-analysis, involving 10,626 cases and 13,592 controls. Of the 19 articles, studies including different subpopulations were considered as separate studies. Consequently, these groups were independently analyzed. Ten studies reported on -1082G/A, nineteen studies on -819C/T, thirteen studies on −592C/A, thirteen studies on rs1518111G/A and three studies on rs1554286C/T. The baseline characteristics of the eligible studies are described in Table 1.

**Table 1:** Characteristics of studies included in the meta-analysis

Author(year)	Ethnicity	Sample size case/control	Mean-age		Genotyping methods	Studied polymorphism	HWE P_value_ (control)	ref
			Case	Control				
Baris S(2016)	Turkish	71/70	37.8 ± 1.0	–	PCR-RFLP	rs1800896	–	27
Hu J. stage I(2015)	Chinese Han	300/350	33.60 ± 8.78	39.66 ± 11.19	PCR-RFLP	rs1800896, rs1800872, rs1554286	*p* > 0.05	28
Hu J. stage II(2015)	Chinese Han	418/1403	33.60 ± 8.78	39.66 ± 11.19	PCR-RFLP	rs1800871	p > 0.05	28
Al-Okaily F(2015)	Saudi	61/211	37.87 ± 12.5	36 ± 10	PCR-ARMS	rs1800896, rs1800871, rs1800872	–	29
Talaat R. M(2014)	Egyptian	87/97	34.37 ± 10.36	–	PCR-SSP	rs1800896, rs1800871	p > 0.05	30
Xavier J. M(2012)	Iranian	973/637	39.1 ± 11.0	42.0 ± 11.6	Sequenom iPlex assay	rs1800896, rs1518111, rs1554286	*P* > 0.01	26
Shahram F(2011)	Iranian	150/140	–	–	PCR-SSP	rs1800896, rs1800871, rs1800872	p > 0.05	31
Ates O(2010)	Turkish	102/102	37.2 ± 8.4	52 ± 7	PCR-ARMS	rs1800896, rs1800871, rs1800872	–	32
Dilek K(2009)	Turkish	97/127	–	–	PCR-SSP	rs1800896, rs1800871, rs1800872	p > 0.05	33
Wallace GR(2007)	UK	63/182	–	–	PCR-SSP	rs1800896, rs1800871	p > 0.05	34
Wallace GR(2007)	ME	115/113	–	–	PCR-SSP	rs1800896, rs1800871	p > 0.05	34
Kramer M(2018)	Turkish	64/68	–	–	Sanger technique	rs1800871, rs1800872, rs1518111	–	35
Kramer M(2018)	Israeli	25/20	–	–	Sanger technique	rs1800871, rs1800872, rs1518111	–	35
Yu H(2017)	Chinese Han	1206/2475	34.86 ± 9.9	35.46 ± 10.3	Sequenom Mass Array	rs1800871, rs1554286	–	36
Carapito R(2015)	Iranian	552/417	–	–	Taqman	rs1800871, rs1518111	–	37
Wu Z(2014)	Chinese	407/679	38.02 ± 12.44	38.81 ± 10.45	Sequenom Mass Array	rs1800871, rs1518111	p > 0.05	38
Khaib Dit Naib O(2013)	Western Algeria	51/96	26 ± 11	–	Direct sequencing	rs1800871, rs1800872	–	39
Kirino Y(2013)	Turkish	1209/1278	–	–	GWAS	rs1800871, rs1518111	*P* < 0.0001	40
Mizuki N(2010)	Japanese	611/737	–	–	GWAS	rs1800871, rs1800872	P < 0.001	7
Mizuki N(2010)	Korean	124/140	–	–	GWAS	rs1800871, rs1800872	P < 0.001	7
Mizuki N(2010)	Turkish	1215/1279	–	–	GWAS	rs1800871, rs1800872	P < 0.001	7
Afkari B(2018)	Iranian	47/58	38.02 ± 10.25	37.4 ± 8.5	PCR-RFLP	rs1800872	p > 0.05	41
Montes-Cano MA(2013)	Spanish	304/313	38.7 ± 13.8	–	Taqman	rs1800872	p > 0.05	42
Remmers EF(2010)	Discovery-Turkish	1161/1221	–	–	GWAS	rs1518111	*p* > 0.00001	8
Remmers EF(2010)	Rplication-Turkish	110/224	–	–	GWAS	rs1518111	p > 0.00001	8
Remmers EF(2010)	Replication-Middle Eastern Arab	188/163	–	–	GWAS	rs1518111	p > 0.00001	8
Remmers EF(2010)	Replication-Greek	107/84	–	–	GWAS	rs1518111	p > 0.00001	8
Remmers EF(2010)	Replication-UK Caucasian	120/119	–	–	GWAS	rs1518111	p > 0.00001	8
Remmers EF(2010)	Replication-Korean	77/52	–	–	GWAS	rs1518111	p > 0.00001	8
Remmers EF(2010)	Replication-Japanese	611/737	–	–	GWAS	rs1518111	p > 0.00001	8

#### Association between rs1800896 polymorphism and risk of BD

**Fig. 2:**
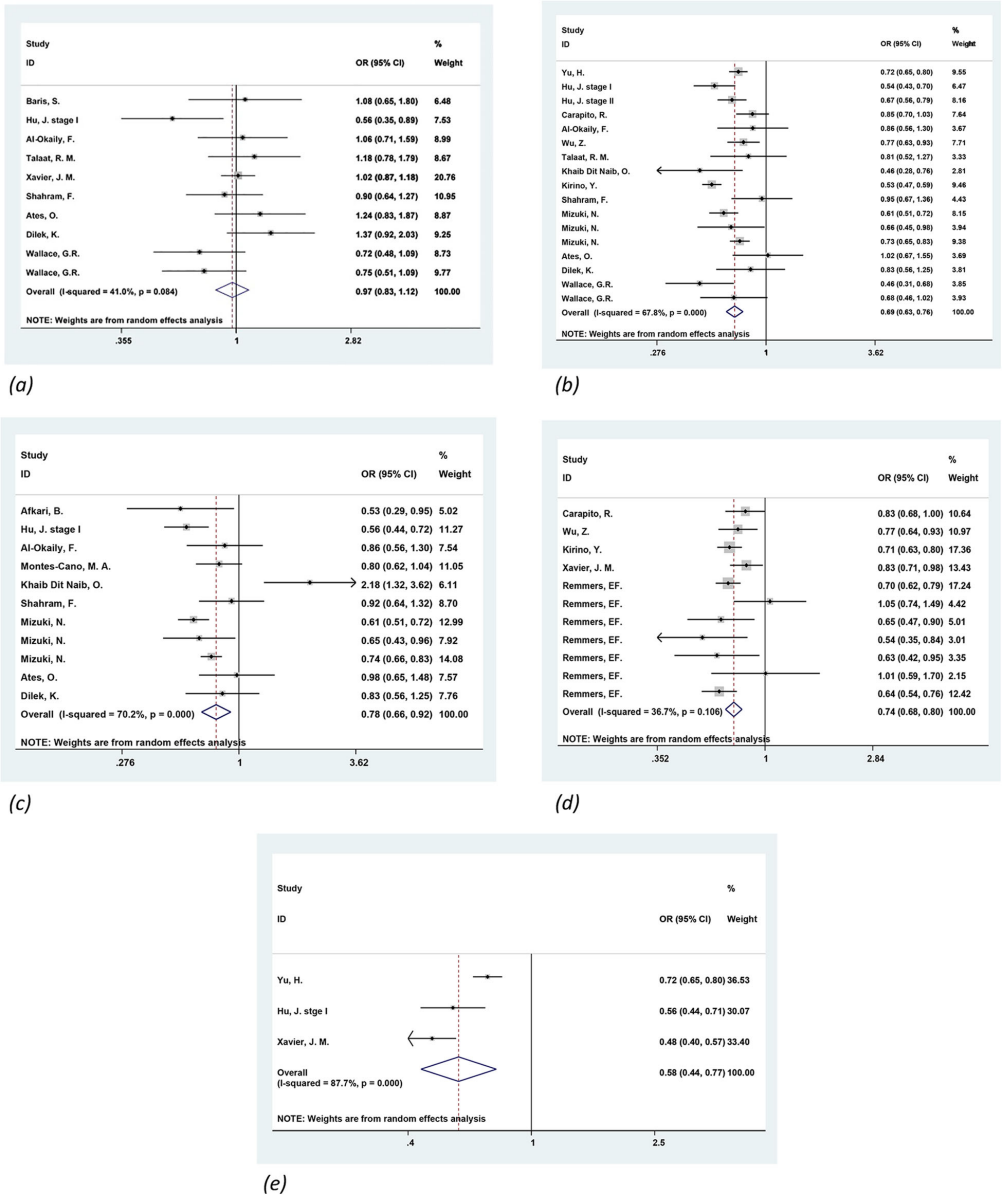
Forest plot of the association of IL-10 polymorphisms with BD. A random-effects model for the OR with 95% CI was used to detect an association between IL-10 polymorphisms with BD. (**a**) rs1800896 (**b**) rs1800871 (**c**) rs1800872 (**d**) rs1518111 (e) rs1554286

Based on ten studies [26–34], including 1970 cases and 1930 controls, the association between IL-10 -1082G/A polymorphism and BD susceptibility was examined. The combined results showed that considering all the studies, the comparisons of allele and genotypes failed to detect any statistical association under the random-effects model (Table 2 and Fig. 2).

**Table 2:** Meta-analysis of the association between IL-10 polymorphisms and BD risk

polymorphism	No. of studies	Comparison	Test of association		Test of heterogeneity			Egger’s test (P)	Metareg’s test (P)
			OR (95% CI)	P-value	P- value	I^2^ (%)	Q test		
rs1800896 A/G	10	GG vs. AA	0.925 (0.684–1.251)	0.613	0.266	19.9	9.98	0.746	0.689
		GG + GA vs. AA	0.936 (0.733–1.195)	0.596	0.020	56/0	18.20	0.836	0.866
		GG vs. GA + AA	0.975 (0.660–1.441)	0.900	0.025	54.4	17.55	0.619	0.640
		AG vs. AA	0.945 (0.717–1.245)	0.687	0.007	62.2	21.17	0.822	0.870
		G vs. A	0.965 (0.831–1.120)	0.640	0.084	41.0	15.25	0.816	0.807
rs1800871 T/C	19	CC vs. TT	0.466 (0.368–0.589)	0.000	0.003	57.4	32.88	0.081	0.347
		CC + CT vs. TT	0.692 (0.584–0.820)	0.000	0.000	66.1	41.33	0.842	0.674
		CC vs. CT + TT	0.557 (0.405–0.767)	0.000	0.000	86.6	119.02	0.032	0.123
		CT vs. TT	0.842 (0.572–1.240)	0.384	0.000	93.7	220.52	0.542	0.883
		C vs. T	0.691 (0.626–0.762)	0.000	0.000	67.8	49.73	0.363	0.493
rs1800872 A/C	13	CC vs. AA	0.723 (0.440–1.186)	0.199	0.000	72.4	32.63	0.161	0.360
		CC + CA vs. AA	0.713 (0.535–0.951)	0.021	0.004	62.4	23.94	0.116	0.276
		CC vs. CA + AA	0.776 (0.583–1.032)	0.082	0.004	60.3	27.71	0.210	0.367
		CA vs. AA	0.716 (0.546–0.940)	0.016	0.024	53.0	19.13	0.101	0.313
		C vs. A	0.779 (0.662–0.915)	0.002	0.000	70.2	33.59	0.194	0.423
rs1518111 A/G	13	GG vs. AA	0.570 (0.471–0.690)	0.000	0.459	0.0	1.56	0.622	0.741
		GG + GA vs. AA	0.697)0.596–0.814)	0.000	0.393	0.0	1.87	0.684	0.533
		GG vs. GA + AA	0.701 (0.623–0.790)	0.000	0.505	0.0	3.32	0.558	0.967
		AG vs. AA	0.786 (0.667–0.927)	0.004	0.723	0.0	0.65	0.604	0.645
		G vs. A	0.738 (0.681–0.800)	0.000	0.106	36.7	15.79	0.500	0.352
rs1554286 T/C	3	CC vs. TT	0.508 (0.372–0.694)	0.000	0.198	38.2	3.23	0.795	0.868
		CC + CT vs. TT	0.605)0.467–0.785(	0.000	0.087	59.1	4.89	0.626	0.466
		CC vs. CT + TT	0.665)0.484–0.916(	0.012	0.041	68.7	6.40	0.487	0.893
		CT vs. TT	0.646 (0.502–0.832)	0.001	0.115	53.8	4.33	0.656	0.506
		C vs. T	0.582 (0.440–0.770)	0.000	0.000	87.7	16.23	0.384	0.265

#### Association between rs1800871 polymorphism and risk of BD

In total, we identified nineteen studies [7, 28–40] which contained 6854 cases and 9911 controls assessed the effect of the IL-10 -819C/T polymorphism in the occurrence of BD. By using the random-effects model, meta-analysis revealed a significant association between BD and the IL-10 -819C/T polymorphism under the allelic (OR = 0.691, 95% CI: 0.626–0.762, *P* < 0.001), homozygous (OR = 0.466, 95% CI: 0.368–0.589, *P* < 0.001), dominant (OR = 0.692, 95% CI: 0.584–0.820, *P* < 0.001), and recessive (OR = 0.557, 95% CI: 0.405–0.767, *P* < 0.001) models, while no significant association was found under the heterozygous model (*P* = 0.384) (Table 2 and Fig. 2).

#### Association between rs1800872 polymorphism and risk of BD

For IL-10 -592C/A polymorphism, thirteen studies [7, 28, 29, 31–33, 35, 39, 41, 42] were involved composed of 3132 BD patients and 3638 controls. The results of the combined analysis of the association between -592 polymorphism and BD are summarized in Table 2 and Fig. 2. There were significant associations for C vs. A allele, CC + CA vs. AA and CA vs. AA genotypes; however, other comparisons failed to obtain any significant association.

#### Association between rs1518111 polymorphism and risk of BD

Thirteen studies [8, 26, 35, 37, 38, 40], including 5519 cases and 5643 controls focused on the relationship between rs1518111 polymorphism and BD risk. The combined results (Table 2 and Fig. 2) showed significant differences in all models between BD cases and healthy controls.

#### Association between rs1554286 polymorphism and risk of BD

In total, three studies [26, 28, 36] which contained 2421 cases and 3429 controls were performed to assess the importance of rs1554286 polymorphism for BD susceptibility. As shown in Table 2 and Fig. 2, this polymorphism was found to be significantly associated with BD susceptibility under all models.

### Heterogeneity and publication bias

**Fig. 3:**
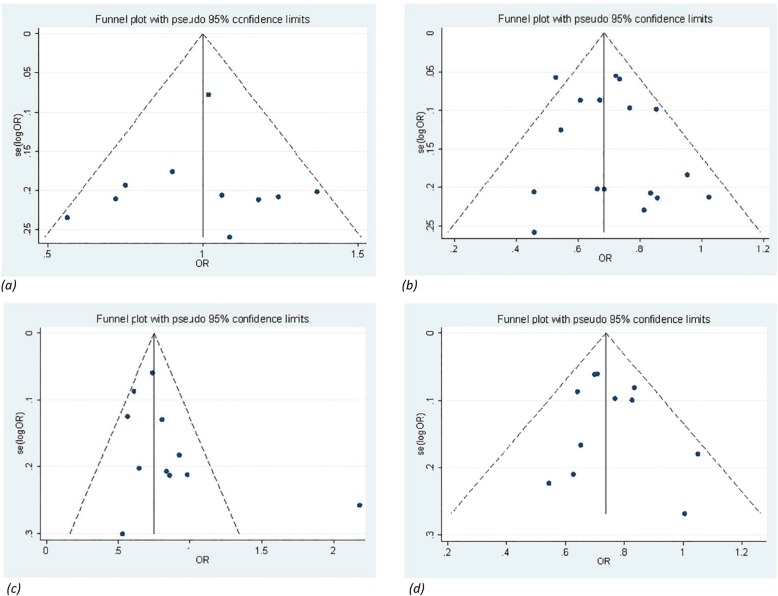
Begg’s funnel plot for publication bias analysis of the IL-10 polymorphisms with BD. Symmetry in Begg’s funnel plots demonstrate the absence of publication bias in the studies investigating the association of (**a**) rs1800896 (**b**) rs1800871 (**c**) rs1800872 (**d**) rs1518111

A funnel plot was used to reveal any publication bias influencing the analysis. The funnel plots did not show a significant sign of asymmetry for IL-10 polymorphisms. For rs1554286, a funnel plot was not performed because it is useless when the number of studies is limited (Fig. 3). The Metareg’s test and Egger’s test were also conducted and the findings showed a lack of publication bias among all comparison models except heterozygous (CC vs. CT + TT) model of rs1800871.

To test heterogeneity among the selected studies, the I^2^ statistic was employed. The heterogeneity was significant under the genetic models of CC vs. CT + TT and CT vs. TT of rs1800871. After excluding the study by Kirino [40], the heterogeneity was eliminated (I^2^ = 44.3, association *P* < 0.001) and (I^2^ = 19.6, association *P* < 0.001), respectively. Significant heterogeneity was detected in C vs. T model of rs1554286 polymorphism. It can be due to a small number of studies included in this analysis. Therefore, the random-effects model was applied to synthesize the data (Table 2).

## Discussion

In order to better understand the possible genetic association of the IL-10 gene polymorphisms and BD, we conducted a systematic review and meta-analysis using pooling the results of the independent usable studies to determine whether the IL-10 polymorphisms have a potential impact on the BD susceptibility.

Behçet’s disease is a multi-system inflammatory disorder with complex etiology, therefore genes involved in the immune system and inflammatory responses are potential candidate genes for the BD development. In addition to HLA-B51 as the strongest genetic factor for BD, which explains 20% incidence of BD [43, 44], GWAS and subsequent genetic studies have identified a number of non-HLA potent loci for the susceptibility and organ damages of this disease, such as cytokines (IL-4 [45], IL-6 [46], IL-10 [7, 40], CCL2 [47, 48], TNF-α [49]), ERAP1 [40], UBASH3B [50, 51], STAT4 [52].

Several studies have been conducted on the influence and impression of the interleukin-10 gene and its protein on the probability of the multiple autoimmune and inflammatory diseases, such as BD relying on its chromosomal location and functional relevance.

Research on the effect of IL-10 on various inflammatory models has led to different and sometimes contradictory results even in the same models, indicating that there are intricate influenced agents on the regulation of cellular responses by IL-10. In the experimental study by Rizzo et al. [53] indicated that IL-10 knockout mice had a susceptibility to develop the Experimental Autoimmune Uveoretinitis (EAU) in comparison to controls, also, IL-10-treatment in induced mice for the EAU showed a resistance to the disease. Other animal studies have also confirmed the protective effect of IL-10 on the ocular inflammation and CNS autoimmune [54–56]. Some studies on BD have observed a reduction in IL-10 protein level in BD patients’ peripheral blood and aqueous humor [13, 30, 41, 57], while in other studies, there has been an increase in the concentration of this protein in plasma and active ulcers of BD [58–63]. On the other hand, there is a study that has not recorded significant changes between patients and controls [64]. Similar to the protein concentration determination studies, the results of the mRNA expression level were varied among previous studies. Several investigations [13, 41, 64] have shown low expression of the IL-10 mRNA in B cells and serums of BD patients. In contrast, Ahmed Ben et al. [65] have reported a high expression of the IL-10 mRNA in oral/genital ulcers, pseudofolliculitis lesions and sites of positive pathergy test. In contrast, Balkan et al. [66] found no significant difference in the IL-10 mRNA expression.

So far, different factors have been studied in the production and regulatory the role of IL-10. One of these influential factors that have recently been studied on IL-10 is epigenetic agents. The study of Alipour et al. [13] showed a hyper-methylation on IL-10 gene promoter region. Genetic variants are other factors that account for up to 75% of the IL-10 expression variance in inter-individual human [67]. Investigation of the polymorphic region in the transcriptional regulation of IL-10 gene may be helpful in predicting inflammatory diseases such as BD. Three common polymorphisms of IL-10 (− 1082G/A, − 819C/T and -592C/A) have been evaluated in detail for their impacts on gene transcription. In vitro studies showed that the highest IL-10 expression has been observed in GCC (− 1082/− 819/− 592) haplotype and lowest expression in AT (− 1082/− 819) haplotype [68–70]. However, studies on BD have not confirmed the effects of the above alleles [30]. In addition to the promoter variants, the intronic variant of rs1518111 is also likely to influence transcription [66].

The present meta-analysis was performed to examine the association between the IL-10 polymorphisms (− 1082, − 819, − 592, rs1518111, and rs1554286) and BD susceptibility in different ethnic groups including East Asian, Middle Eastern, European, and African populations. The strongest associations were observed in the comparisons of allele and genotypes of − 819C/T promoter SNP, rs1518111G/A intronic SNP, and rs1554286C/T intronic SNP. The overall results indicated the protective impact of these polymorphisms against the disease. So that, individuals carrying C allele of −819 and rs1554286 have 31 and 42% lower risk of developing BD, respectively. We also achieved significant association in -592C/A promoter SNP, which showed a relatively protective role against BD. However, the comparisons of allele and genotypes of -1082G/A promoter SNP failed to demonstrate any significant association.

However, our results should be interpreted cautiously, with respect to the following limitation reasons. First, limited number of included studies in some studied SNPs; second, heterogeneity and publication bias, for instance, missing articles due to unpublished studies, possibly with negative results, and/or language bias due to writing in other languages (except English and Persian); third, lack of sufficient data on the characteristics and symptoms of the participants in most of the primary studies to perform subgroups analysis, such as based on age, gender and the disease activity; lastly, some genotype distributions of control groups do not follow the HWE.

## Conclusions

Our data demonstrated significant associations of the IL-10 polymorphisms with BD. Accordingly, the polymorphisms of -819, − 592, rs1518111 and rs1554286 play relatively protective roles against Behcet’s disease.

## Methods

### Search strategy

A comprehensive search for the IL-10 polymorphisms and BD was performed in PubMed, Embase, Web of Science, and Scopus databases for articles in English, and SID, Magiran, IranMedex, and IranDoc for articles in Persian up to February 2019 according to Preferred Reporting Items for Systematic Reviews and Meta-Analyses (PRISMA) checklist and PICO approach [43]. The most relevant articles were identified by computerized searches using relevant text words and medical subject headings (MeSH) that included all spellings and combination of “Behcet’s syndrome” or “Behcet’s disease” and “Interleukin-10” or “IL-10”. Besides, we hand-searched reference lists of selected publications and previous meta-analysis study to ensure that all relevant studies and recent reviews were included. The search was limited to case-control studies and Genome-Wide Association Studies (GWAS). No restrictions were placed on race, ethnicity or geographic area publication date.

### Inclusion and exclusion criteria

A study was included in this meta-analysis if (1) it was published by February 2019, (2) it was a case-control study or GWAS that determined the distributions of the IL-10 -1082G/A, −819C/T, −592C/A, rs1518111G/A, and rs1554286C/T polymorphisms, in patients with BD and controls, (3) it contained original data, and (4) it provided enough data to calculate odds ratios (ORs). Exclusion criteria were as follows: (1) Keywords/search terms/reviews, and abstract scanning criteria, (2) the study was a review or abstract and publications in duplicate, (3) unrelated to each of BD and these polymorphisms, (4) studies involving family members, because their analysis was based on linkage considerations, and (5) papers do not involve human subjects.

### Data extraction

Two authors (E.S. and N.R.) independently extracted the following data from all studies selected by using a study-specific data extraction form: first author’s name, year of publication, ethnicity, mean age of participants, number of cases and controls and method for determination of genotype, studied polymorphisms, Hardy–Weinberg equilibrium (HWE) *P*-values. Any disagreement between authors was resolved by discussion until a consensus was reached. If they failed to reach an agreement, a third author (M.G.) was consulted to resolve the discrepancies. Due to the lack of the mean age and HWE *P*-values for some studies, we were not able to analyze the data based on this information. This study was conducted in the Liver and Gastrointestinal Disease Research Center, Tabriz University of Medical Sciences.

### Statistical analyses

All statistical manipulations were performed with STATA version-15.0 software (STATA Corporation, College Station, Texas). The strength of the association between IL-10 polymorphisms and BD was assessed by calculating pooled OR and 95% CI values in the following genetic models: allelic model (G vs. A, C vs. A, C vs. T), dominant model (GG + GA vs. AA, CC + CT vs. TT, CC + CA vs. AA), recessive model (GG vs. GA + AA, CC vs. CT + TT, CC vs. CA + AA), homozygous model (GG vs. AA, CC vs. TT, CC vs. AA) and heterozygous model (AG vs. AA, CT vs. TT, CA vs. AA). Z-test with *P* < 0.05 was used to authenticate the statistical significance of effect size. Sensitivity analysis was used to investigate the cause of dispersion. Then, outlier studies were removed and analysis was recomputed. In the case of a significant reduction in dispersion (I^2^ index), this study was considered as a dispersion factor. With regard to the heterogeneity of the studies, Cochran’s Q test (*p*-value [phet] < 0.10 was considered as statistically significant heterogeneity) and I^2^ statistics (75 ≤ I^2^ < 100 as extreme heterogeneity, 50 ≤ I^2^ < 75 as high heterogeneity, 25 ≤ I^2^ < 50 as moderate heterogeneity, and I^2^ < 25 as no heterogeneity) were used to assess the degree of heterogeneity [71]. The random-effects model was used for meta-analysis because it accounts for random variability both within and among studies. Publication bias was investigated by Funnel plot and Egger’s test. A *P-value* < 0.05 was considered statistically significant for all analyses.

## Data Availability

The datasets used and/or analyzed during the current study are available from the corresponding and first authors on reasonable request.

## Abbreviations

BD: Behcet’s disease
CI: Confidence Interval
EAU: Experimental Autoimmune Uveoretinitis
GWAS: Genome-Wide Association Study
HWE: Hardy–Weinberg equilibrium
IL-10: Interleukin-10
LD: linkage disequilibrium
OR: Odds Ratio
SNP: Single Nucleotide Polymorphism
